# The relationship between breastfeeding and reported respiratory and gastrointestinal infection rates in young children

**DOI:** 10.1186/s12887-019-1693-2

**Published:** 2019-09-18

**Authors:** Nicole M. Frank, Kristian F. Lynch, Ulla Uusitalo, Jimin Yang, Maria Lönnrot, Suvi M. Virtanen, Heikki Hyöty, Jill M. Norris, for the TEDDY Study Group, Marian Rewers, Kimberly Bautista, Judith Baxter, Daniel Felipe-Morales, Kimberly Driscoll, Brigitte I. Frohnert, Marisa Gallant, Patricia Gesualdo, Michelle Hoffman, Rachel Karban, Edwin Liu, Jill Norris, Adela Samper-Imaz, Andrea Steck, Kathleen Waugh, Hali Wright, Jorma Toppari, Olli G. Simell, Annika Adamsson, Suvi Ahonen, Heikki Hyöty, Jorma Ilonen, Sanna Jokipuu, Leena Karlsson, Miia Kähönen, Mikael Knip, Mirva Koreasalo, Kalle Kurppa, Tiina Latva-aho, Maria Lönnrot, Markus Mattila, Elina Mäntymäki, Katja Multasuo, Tiina Niininen, Sari Niinistö, Mia Nyblom, Paula Ollikainen, Petra Rajala, Jenna Rautanen, Anne Riikonen, Minna Romo, Suvi Ruohonen, Juulia Rönkä, Satu Simell, Tuula Simell, Maija Sjöberg, Aino Stenius, Sini Vainionpää, Eeva Varjonen, Riitta Veijola, Suvi M. Virtanen, Mari Vähä-Mäkilä, Mari Åkerlund, Katri Lindfors, Jin-Xiong She, Desmond Schatz, Diane Hopkins, Leigh Steed, Jennifer Bryant, Janey Adams, Katherine Silvis, Michael Haller, Melissa Gardiner, Richard McIndoe, Ashok Sharma, Stephen W. Anderson, Laura Jacobsen, John Marks, Anette G. Ziegler, Andreas Beyerlein, Ezio Bonifacio, Anita Gavrisan, Cigdem Gezginci, Anja Heublein, Michael Hummel, Sandra Hummel, Annette Knopff, Charlotte Koch, Sibylle Koletzko, Claudia Ramminger, Roswith Roth, Marlon Scholz, Joanna Stock, Katharina Warncke, Lorena Wendel, Christiane Winkler, Åke Lernmark, Daniel Agardh, Carin Andrén Aronsson, Maria Ask, Jenny Bremer, Ulla-Marie Carlsson, Corrado Cilio, Emelie Ericson-Hallström, Annika Fors, Lina Fransson, Thomas Gard, Rasmus Bennet, Carina Hansson, Susanne Hyberg, Hanna Jisser, Fredrik Johansen, Berglind Jonsdottir, Silvija Jovic, Helena Elding Larsson, Marielle Lindström, Markus Lundgren, Maria Månsson-Martinez, Maria Markan, Jessica Melin, Zeliha Mestan, Caroline Nilsson, Karin Ottosson, Kobra Rahmati, Anita Ramelius, Falastin Salami, Sara Sibthorpe, Anette Sjöberg, Birgitta Sjöberg, Carina Törn, Anne Wallin, Åsa Wimar, William A. Hagopian, Michael Killian, Claire Cowen Crouch, Jennifer Skidmore, Ashley Akramoff, Jana Banjanin, Masumeh Chavoshi, Kayleen Dunson, Rachel Hervey, Rachel Lyons, Arlene Meyer, Denise Mulenga, Jared Radtke, Davey Schmitt, Julie Schwabe, Sarah Zink, Dorothy Becker, Margaret Franciscus, Mary Ellen Dalmagro-Elias Smith, Ashi Daftary, Mary Beth Klein, Chrystal Yates, Jeffrey P. Krischer, Sarah Austin-Gonzalez, Maryouri Avendano, Sandra Baethke, Rasheedah Brown, Brant Burkhardt, Martha Butterworth, Joanna Clasen, David Cuthbertson, Christopher Eberhard, Steven Fiske, Dena Garcia, Jennifer Garmeson, Veena Gowda, Kathleen Heyman, Belinda Hsiao, Francisco Perez Laras, Hye-Seung Lee, Shu Liu, Xiang Liu, Kristian Lynch, Colleen Maguire, Jamie Malloy, Cristina McCarthy, Aubrie Merrell, Steven Meulemans, Hemang Parikh, Ryan Quigley, Cassandra Remedios, Chris Shaffer, Laura Smith, Susan Smith, Noah Sulman, Roy Tamura, Ulla Uusitalo, Kendra Vehik, Ponni Vijayakandipan, Keith Wood, Jimin Yang, Liping Yu, Dongmei Miao, Polly Bingley, Alistair Williams, Kyla Chandler, Olivia Ball, Ilana Kelland, Sian Grace, Ben Gillard, William Hagopian, Sandra Ke, Niveen Mulholland, Beena Akolkar, Kasia Bourcier, Thomas Briese, Suzanne Bennett Johnson, Eric Triplett

**Affiliations:** 1 University of Virginia Children’s Hospital, Charlottesville, VA USA; 2 Health Informatics Institute, Morsani College of Medicine, University of South Florida, Tampa, FL USA; 3 Faculty of Medicine and Health Technology, University of Tampere, Tampere, Finland; 4 National Institute for Health and Welfare, Helsinki, Finland; 5 School of Health Sciences, University of Tampere, Tampere, Finland; 6 Center for Child Health Research, University of Tampere and Tampere University Hospital, Tampere, Finland; 7 The Science Center of Pirkanmaa Hospital District, Tampere, Finland; 8 Fimlab Laboratories, Pirkanmaa Hospital District, Tampere, Finland; 9 Department of Epidemiology, University of Colorado Denver, Colorado School of Public Health, Aurora, CO USA

**Keywords:** Breastfeeding, Infection, Illness, Gastroenteritis, Gastrointestinal, Respiratory, Otitis media

## Abstract

**Background:**

Although breastfeeding is touted as providing many health benefits to infants, some aspects of this relationship remain poorly understood.

**Methods:**

The Environmental Determinants of Diabetes in the Young (TEDDY) is a prospective longitudinal study that follows children from birth through childhood, and collects data on illness events, breastfeeding duration, and time to introduction of formula or foods at 3 month intervals up until 4 years of age and at 6 months intervals thereafter. Exclusive and non-exclusive breastfeeding is examined in relation to the 3-month odds of a respiratory or gastrointestinal infection for 6861 children between the ages of 3–18 months, and 5666 children up to the age of 4 years. Analysis was performed using logistic regression models with generalized estimating equation methodology. All models were adjusted for potential confounding variables.

**Results:**

At 3–6 months of age, breastfeeding was found to be inversely associated with the odds of respiratory infections with fever (OR = 0.82, 95% CI = 0.70–0.95), otitis media (OR = 0.76, 95% CI = 0.62–0.94), and infective gastroenteritis (OR = 0.55, 95% CI = 0.46–0.70), although the inverse association with respiratory illnesses was observed only for girls during the winter months. Between 6 and 18 months of age, breastfeeding within any 3 month period continued to be inversely associated with the odds of ear infection and infective gastroenteritis, and additionally with the odds of conjunctivitis, and laryngitis and tracheitis, over the same 3 month period within this age range. However, breastfeeding in this group was associated with increased reports of common cold. Duration of exclusive breastfeeding was inversely associated with the odds of otitis media up to 48 months of age (OR = 0.97, 95% CI = 0.95–0.99) after breastfeeding had stopped.

**Conclusions:**

This study demonstrates that breastfeeding can be protective against multiple respiratory and gastrointestinal acute illnesses in some children up to at least 6 months of age, with duration of exclusive breastfeeding being somewhat protective of otitis media even after breastfeeding has stopped.

**Trial registration:**

ClinicalTrials.gov Identifier: NCT00279318.

Date of registration: January 17, 2006 (proactively registered).

First Posted: January 19, 2006.

## Background

The World Health Organization (WHO) recommends that babies be exclusively breastfed until the age of 6 months, and continue to receive breast milk supplementary to solid foods for up to 2 years or beyond [1]. The medical communities in the United States and in Europe echo this recommendation with similar guidelines, recommending exclusive breastfeeding for the first 4 to 6 months of a baby’s life [2–4]. One driving force behind this policy is the mounting evidence of a multitude of health benefits to the child as a result of breastfeeding. The Environmental Determinants of Diabetes in the Young (TEDDY) study is a large international observational study following children from birth throughout childhood. Among other information related to environmental exposures, this study records initiation and cessation of breastfeeding, timing of introduction to other foods, and all illness events for each participant. As such, it is well-placed to add to existing literature by exploring the relationship between breastfeeding and rates of acute illnesses, both during the time of breastfeeding and beyond.

The health benefits of breastfeeding can be thought of as belonging to two categories – immediate benefits and future benefits. Immediate benefits are those benefits that a child receives from breastmilk during the time they are breastfed. For example, during the time period when breastfeeding is occurring, past studies have found a correlation between breastfeeding and reduced frequency of otitis media episodes [5–8], gastrointestinal infections [8–11], lower respiratory infections [10–15], upper respiratory infections [11–14], urinary tract infections [16–18], illness events in general [10, 19], and hospitalizations [19, 20]. However, for respiratory and gastrointestinal infections, in particular, there continues to be some uncertainty about whether only exclusive breastfeeding is protective [11, 14], any breastfeeding is protective [8, 10, 12, 13, 15], or if breastfeeding is perhaps not protective at all [8]. Therefore, although these relationships have been studied, the lack of consensus in previous studies’ results leaves a gap in our understanding of the interplay between breastfeeding and concurrent respiratory and gastrointestinal illness in children. This paper provides new evidence that weighs in on some of these contested findings.

Future benefits from breastfeeding, on the other hand, are those that persist, or even manifest, after breastfeeding has stopped. The majority of studies examining future benefits of breastfeeding focus on various chronic non-communicable diseases. In this vein, breastfeeding as an infant has been shown to be associated with reduced risk of obesity, cardiovascular disease, diabetes, cancer, and atopic disease (like asthma) later in life [21–24]. Considerably less research has focused on the impact of breastfeeding on acute illnesses in early childhood. Some studies that have explored this topic have suggested a reduced rate of otitis media [25–29], respiratory infections [26–28, 30–32], throat infections [29], sinus infections [29], and hospitalizations [20] among children who were breastfed early in life. This paper will add new insight into the poorly understood relationship between breastfeeding as an infant and rates of acute illness during early childhood. More specifically, it will look at whether exclusive breastfeeding for longer duration has a larger impact on future rates of acute respiratory and gastrointestinal illness during childhood than exclusive breastfeeding for shorter duration.

## Methods

The Environmental Determinants of Diabetes in the Young (TEDDY) is a prospective cohort study funded by the National Institutes of Health with the primary goal to identify environmental causes of type 1 diabetes (T1D). It includes six clinical research centers - three in the US: Colorado, Georgia/Florida, Washington; and three in Europe: Finland, Germany, and Sweden. Children at each location were screened at birth for high risk genes for T1D, and those found to have these genetic markers were invited to be followed by the TEDDY Study. Detailed study design and methods have been previously published [33, 34]. Written informed consents were obtained for all study participants from a parent or primary caretaker for genetic screening and, separately, for participation in prospective follow-up. The study was approved by local Institutional Review Boards and is monitored by the External Evaluation Committee formed by the National Institutes of Health.

Study participants come to the clinic every 3 months until the age of 4 years, and then every 6 months beyond this. Because the interval of reporting changes at the age of 4 years, for the purpose of this study, only follow-up data up to the age of 4 years will be included in the analysis. Between visits, parents record a wide variety of environmental exposures – including detailed information on breastfeeding, diet, and illness events – in a TEDDY book, or log, which is then reviewed by a clinician at each visit. The clinician uses this log, as well as parental input, to complete a questionnaire, which compiles the information from the TEDDY book in an organized fashion, and which is the source of the data used in this analysis. Therefore, the age at which the mother stopped exclusively breastfeeding (or introduced other foods or formulas), and the age at which the mother stopped breastfeeding at all were gleaned from these clinic visit questionnaires. Likewise, the TEDDY book (and therefore the visit questionnaires) records all reported infections experienced by the child since the last visit. The data collector reviews the reported illness symptoms, asks for clarifying information when appropriate, and assigns an ICD code or codes to each infectious episode, which are then entered into the study database. These codes are extracted from the database for data analysis. TEDDY has developed a method for reporting and classifying acute infectious diseases using these self-reported data, which is described in a previous publication [35].

The longitudinal follow-up of the children was broken up into three-month age intervals, according to time between visits, for each family. The exact intervals were from (but not including) the day of the last visit when the TEDDY book questionnaire was completed, to (and including) the day of the current visit. If the family did not fill in the TEDDY book for a scheduled visit, the intervals were maintained in a 3 month time series by using the expected due date for the visit. All analysis was made on a 3 month level scale to reduce the influence of possible recall bias, or systematic error resulting from differences in accuracy of reporting, as to when an infection occurred within the last 3 months.

**Fig. 1:**
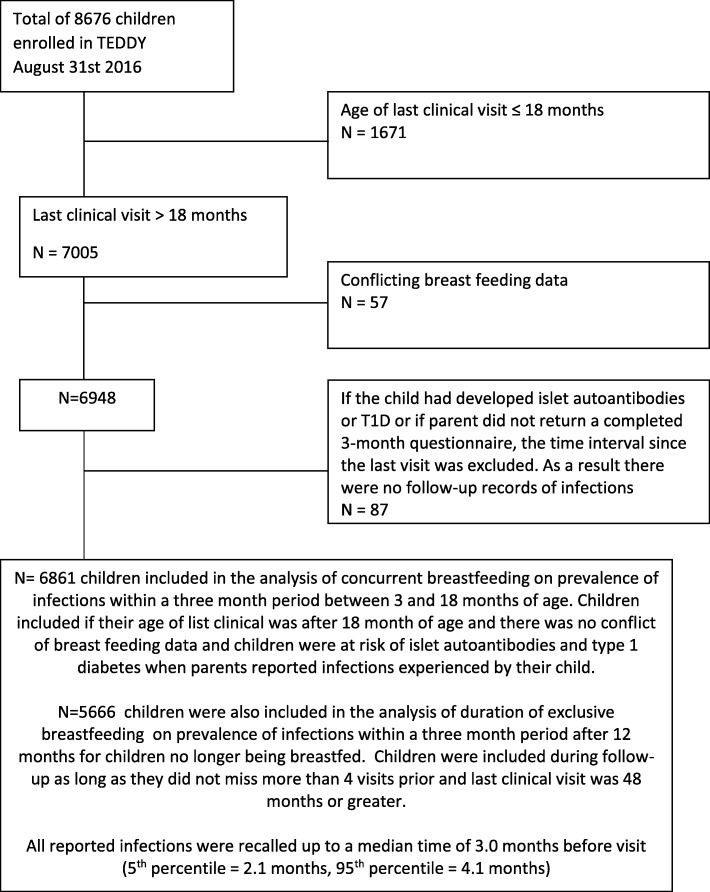
Selection of study population

Of the 8676 children enrolled in TEDDY, 6861 were older than 18 months of age at their last clinic visit, had not developed islet autoantibodies or T1D at the time of questionnaire submission, and had no conflicting reported breastfeeding data (for example, where two different questionnaires listed two different dates of cessation of breastfeeding for the same participant) (Fig. 1). The participants for this analysis were born between September 1st 2004 and February 28th 2010 and were followed up to 48 months of age. We analyzed the data as of August 31st 2016, 2 years after the last 48 month visit window for the study ended.

Data collected after a study participant tested positive for islet autoantibodies or developed T1D were excluded from this analysis due to concern that parents of this group may systematically report illness events differently, thus introducing a source of recall bias to the analysis.

The odds of infections in a three-month period among children who were breastfed at the last visit (i.e. who were breastfeeding at the start of the three-month interval) compared to children who were not breast fed at the last visit (or not breast fed at the start of the three-month interval) were calculated from coefficients of marginal logistic regression models. To account for the correlation of infections reported by the same family at multiple visits, the logistic models were estimated using Generalized Estimating Equations (GEE) with robust standard errors. The mean relationship between breastfeeding and its association with the presence of an infection was of most importance, and, therefore, we compared the coefficients from models assuming an independent, exchangeable and autoregressive covariance structure to make sure careful modeling of the covariance structure was not necessary. Final models used an exchangeable covariance structure, and were adjusted for gender, age of child, age of mother at birth, maternal education, single child, number of rooms in household, parental working and smoking status when child was 9 months of age, country, if the child was a first degree relative of a type 1 diabetic individual, whether daycare or social group had started at the last visit, and season of the year when the 3 month history of infections was reported.

Children who were a) exclusively breastfed (i.e. had not yet been introduced to formula or foods other than breast milk), b) breastfed but not exclusively (i.e. still received breast milk, but had also been introduced to formula and/or other foods) and c) no longer breastfed at 3 months of age were first examined in relation to respiratory and gastrointestinal infections between 3 and 6 month of age. Overall significance of association with each infection was examined by a Wald test. Next, breastfeeding after 6 months, which consisted mostly of children who were non-exclusively breastfed, was examined with relationship to odds of infection at 3 months intervals up to 18 months of age when 93% of children had stopped breastfeeding. Lastly, the relationship between the total duration of exclusive breastfeeding and the prevalence of infections after 12 months of age was examined, adjusted for age of the child and duration of non-exclusive breastfeeding. Of particular interest were associations of breastfeeding with respiratory and gastrointestinal infectious episodes overall, as well as common subsets of respiratory and gastrointestinal infections, including: respiratory infections with fever, common cold, laryngitis and tracheitis, influenza, enterovirus, tonsillitis or Streptococcal pharyngitis, infections of the middle ear, bronchitis and lower respiratory infections, conjunctivitis, gastrointestinal infections with fever, infective gastroenteritis, and gastrointestinal symptoms. Other less common illness categories were excluded. No adjustment for multiple comparisons was made. All *P*-values were two sided. SAS 9.3 (SAS Institute Inc., Cary, NC) was used for the statistical analyses and GraphPad PRISM 5.03 (GraphPad Software Inc., San Diego, CA) for graphs. The STROBE (Strengthening the Reporting of Observational studies in Epidemiology) guidelines were followed in the reporting of this research.

## Results

The cohort included 6861 children who were followed for longer than 18 months, and up to a maximum of 48 months of age, and who were not islet autoantibody positive or diabetic at the time of data collection. In all there were 21,330 person years of follow-up of reported infections with a median recall period of 3 months (Fig. 1). The study population is described in more detail in Table 1.

**Table 1:** Description of study population (*n* = 6861)

Characteristic	N (%)
Sex	
Male	3504 (51.1)
Female	3357 (48.9)
Country	
US-Colorado	1110 (16.2)
US-Georgia/Florida	653 (9.5)
US-Washington	1001 (14.6)
US-satellite sites	7 (0.1)
Germany	436 (6.4)
Finland	1557 (22.7)
Sweden	2097 (30.6)
Maternal Education	
Basic Primary	1252 (18.7)
High School	1648 (24.6)
Beyond High School	3800 (56.7)
missing	161
Age of Mother at Birth	
< 25 years	763 (11.1)
25–29 years	1997 (29.1)
30–34 years	2470 (36.0)
> 35 years	1631 (23.8)
Number of room in house residence	
≤ 3 rooms	780 (11.7)
3–6 rooms	3641 (54.4)
> 6 rooms	2269 (33.9)
missing	171
Maternal Working Status at 9 months	
Working	2385 (35.7)
Not Working	4289 (64.3)
missing	187
Maternal Smoking Status at 9 months	
Smoker	618 (9.2)
Non-smoker	6092 (90.8)
missing	151
Age Child Started Daycare or joined social group	
< 3 months	2065 (30.7)
3–6 months	1647 (24.5)
6–12 months	1266 (18.8)
12–18 months	920 (13.7)
≥ 18 months or no	839 (12.5)
missing	124
Child Birthweight	
SGA (< 2500 g)	221 (3.3)
AGA (2500–4000 g)	5369 (90.2)
LGA (> 4000 g)	1102 (16.5)
missing	169
First Degree Relative with Type 1 Diabetes	
Yes	798 (11.6)
No	6063 (88.4)
Single child in house at 9 months of age	
Yes	2806 (41.9)
No	3896 (58.1)
missing	159
Breastfeeding Duration	
< 3 months	1724 (25.2)
3–6 months	834 (12.2)
6–9 months	1420 (20.8)
9–12 months	1299 (19.0)
12–15 months	726 (10.6)
15–18 months	335 (4.9)
> 18 months	495 (7.3)
missing	28
Exclusive Breastfeeding Duration	
< 3 months	5201 (75.8)
3–6 months	1371 (20.0)
6–9 months	287 (4.2)
9–12 months	2 (0.0)

### Are exclusive and non-exclusive breastfeeding associated with presence of infection between age 3 and 6 months?

**Fig. 2:**
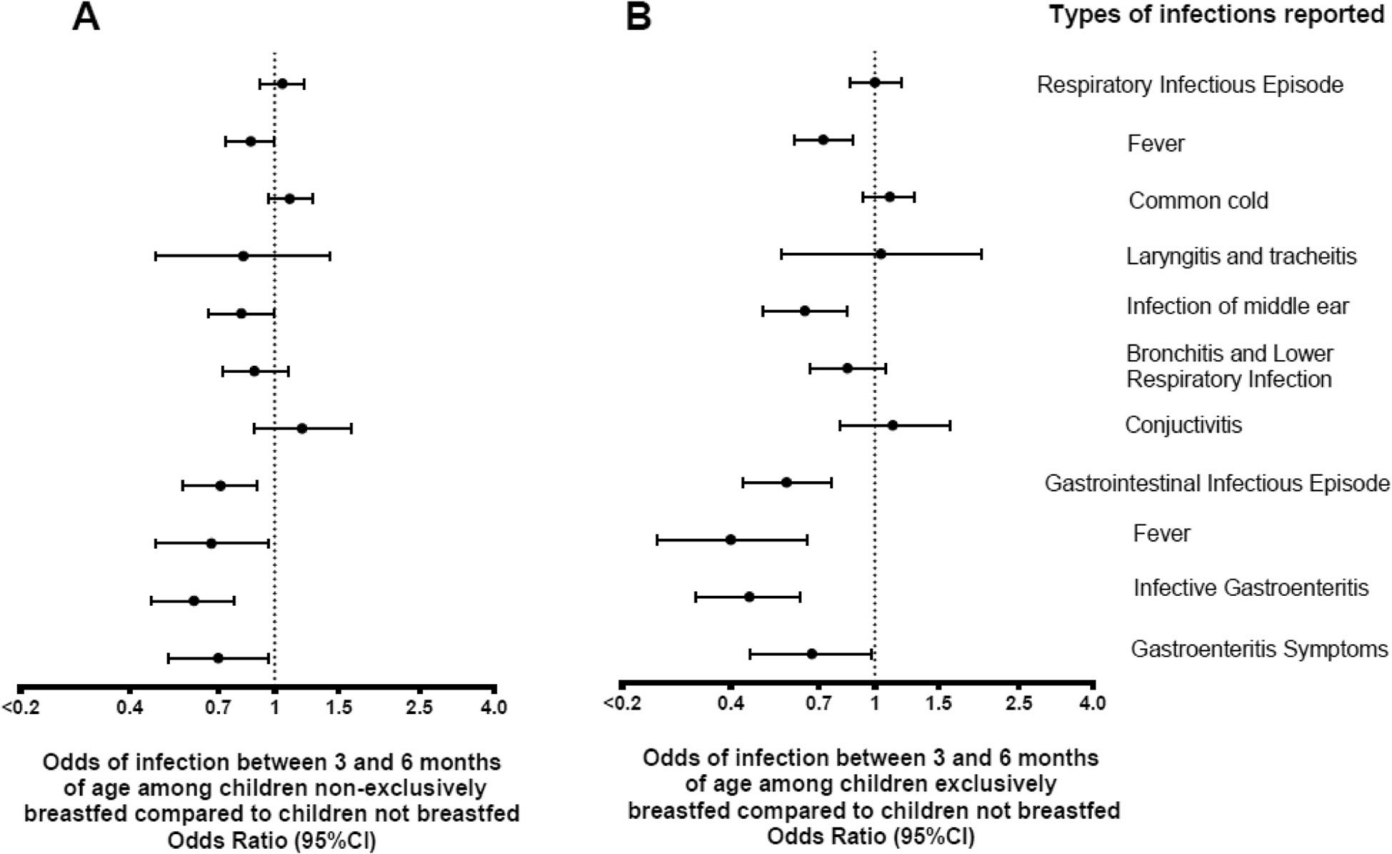
Odds of infection among breastfed children age 3–6 months compared to non-breastfed children

At 6 months of age, 6720/6861 (98.0%) of the children had a parent report of experiencing at least one infection since enrollment at 3 months of age. Of these children 1628 (24.3%) were still being breastfed exclusively at enrollment, 3396 (50.7%) were still breastfed but not exclusively (ie, the breast milk was supplemented with other types of food or formula prior to enrollment), and 1669 (24.9%) were not being breastfed at time of enrollment. The odds of a gastrointestinal infectious episode (*p* = 0.0001) were significantly reduced among children who were breastfed (both exclusively and non-exclusively) compared to children who were not breastfed (Fig. 2). This inverse association was strongest on the odds of gastrointestinal infectious episodes, when the episode included an ICD10 report for infective gastroenteritis (as opposed to just reporting non-specific gastroenteritis symptoms such as nausea or vomiting) (non-exclusive vs no breastfeeding; OR 0.60, 95% CI = 0.46–0.77; exclusive vs no breastfeeding, OR = 0.45, 95% CI = 0.32–0.62). An inverse association was also observed on respiratory infectious episodes with a reported fever (non-exclusive vs no breastfeeding; OR 0.86, 95% CI = 0.73–1.00; exclusive vs no breastfeeding, OR = 0.72, 95% CI = 0.60–0.87) or with a reported otitis media (non-exclusive vs no breastfeeding; OR 0.81, 95% CI = 0.66–1.00; exclusive vs no breastfeeding, OR = 0.64, 95% CI = 0.49–0.84) for both groups (Fig. 2).

### Is breastfeeding associated with presence of infection between 6 and 18 months of age?

Between the age of 6 and 18 months, breastfeeding within any 3 month period remained inversely associated with the odds of otitis media (OR = 0.89, 95% CI = 0.82–0.97, *p* = 0.008) and infective gastroenteritis (OR = 0.89, 95% CI = 0.81–0.98, *p* = 0.01) over the same 3 month period within this age range, but not of febrile respiratory and gastrointestinal episodes in general (Table 2). In addition, for children aged 6 to 18 months, breastfeeding within any 3 month period was more inversely associated with the odds of conjunctivitis (OR = 0.86, 95% CI = 0.74–1.0, *p* = 0.04) and laryngitis and tracheitis (OR = 0.79, 95% CI = 0.63–0.97, *p* = 0.03) over the same 3 month period, compared to children aged 3 to 6 months, for whom no association was found (conjunctivitis: OR = 1.17, 95% CI = 0.87–1.57, *p* = 0.29; laryngitis and tracheitis: OR = 0.88, 95% CI = 0.52–1.49, *p* = 0.64) (Table 2). Of note, when analyzed in smaller age increments (i.e. 6–12 months and 12–18 months), the above trends remained the same (data not shown).

**Table 2:** Breastfeeding in relation to the age-specific odds of an infection within a 3-month period

Infection/symptoms	Age period 3 to 6 months		Age period > 6 to 18 months	
	Breastfed Yes vs. No a-OR (95% CI)		Breastfed Yes vs. No a-OR (95% CI)	*p*-value
Respiratory	1.04 (0.91–1.19)	0.61	**1.17 (1.09–1.26)**	**< 0.0001**
With fever	**0.82 (0.70–0.95)**	**0.008**	**1.08 (1.01–1.15)**	**0.04**
Common cold	1.10 (0.97–1.26)	0.15	**1.25 (1.17–1.34)**	**< 0.0001**
Laryngitis and tracheitis	0.88 (0.52–1.49)	0.64	**0.79 (0.63–0.97)**	**0.03**
Influenza	–		0.85 (0.63–1.15)	0.29
Enterovirus	–		0.97 (0.71–1.33)	0.87
Tonsillitis or Streptococcal pharyngitis	–		1.02 (0.76–1.37)	0.91
Infection of the middle ear	**0.76 (0.62–0.94)**	**0.01**	**0.89 (0.82–0.97)**	**0.008**
Bronchitis and lower resp. infection	0.87 (0.71–1.06)	0.17	1.00 (0.91–1.10)	0.93
Conjunctivitis	1.17 (0.87–1.57)	0.29	**0.86 (0.74–1.00)**	**0.04**
Gastrointestinal	**0.66 (0.53–0.83)**	**0.0003**	0.96 (0.88–1.05)	0.96
With fever	**0.58 (0.41–0.82)**	**0.0002**	0.91 (0.79–1.03)	0.14
Infective gastroenteritis	**0.55 (0.46–0.70)**	**< 0.0001**	**0.89 (0.81–0.98)**	**0.01**
Gastroenteritis symptoms	**0.69 (0.51–0.93)**	**0.02**	1.05 (0.94–1.16)	0.40

a-ORs = odds ratio are adjusted (a) for gender, age of child, age of mother at birth, maternal education, single child, number of rooms in household, parental working and smoking status when child was 9 months of age, country, if the child was a first degree relative of a type 1 diabetic individual, whether daycare or social group had started at the last visit, and season of the year at the start of the interval (i.e. last visit). Bold face type indicates statistical significance with a *p*-value < 0.05

Breastfeeding within any 3 month period among children aged 6 to 18 months was associated with increased odds of reported respiratory infectious episodes over the same 3 month period within this age range (OR = 1.17, 95% CI = 1.09–1.26, *p* < 0.0001), particularly when the episodes included a report of a common cold (OR = 1.25, 95% CI = 1.17–1.34, *p* < 0.0001) (Table 2).

### Does length of exclusive breastfeeding alter the change in odds of an infection after breastfeeding has stopped?

By 9 months of age, all but two children had stopped being exclusively breastfed. At the age of 12 months, none of the children were exclusively breastfed. When examining infections after 12 months of age, each additional month of exclusive breastfeeding was associated with slightly reduced odds of an otitis media episode (/month increase duration of exclusive breastfeeding, OR = 0.97, 95% CI = 0.95–0.99, *p* = 0.004) in children for whom breastfeeding had been stopped (Table 3). No other significant association was found between duration of exclusive breastfeeding during the first 12 months and either gastrointestinal or respiratory infectious episodes after 12 months of age (Table 3).

**Table 3:** Duration of exclusive breastfeeding on the odds of a respiratory or gastrointestinal infection in any three-month interval after 12 months of age among children no longer being breastfed and followed in the study until at least 48 months of age (*n* = 5666)

	Length of exclusive Breastfeeding on odds of infection in a 3 month period	
	/ months a-OR (95% CI)	*p*-value
Respiratory		
Overall	1.009 (0.994–1.023)	0.25
With fever	1.004 (0.990–1.017)	0.61
Common cold	1.013 (0.999–1.028)	0.07
Laryngitis and tracheitis	0.985 (0.939–1.033)	0.54
Influenza	1.027 (0.983–1.073)	0.23
Enterovirus	1.002 (0.959–1.048)	0.92
Tonsillitis or Streptococcal pharyngitis	0.996 (0.956–1.035)	0.84
Infection of the middle ear	**0.971 (0.952–0.991)**	**0.004**
Bronchitis and lower resp. infection	0.993 (0.974–1.012)	0.48
Conjunctivitis	0.988 (0.964–1.012)	0.33
Gastrointestinal		
Overall	1.010 (0.996–1.024)	0.16
With fever	0.998 (0.977–1.018)	0.81
Infective gastroenteritis	1.014 (0.997–1.031)	0.11
Gastroenteritis symptoms	1.010 (0.994–1.026)	0.22

Length of exclusive breastfeeding is included in same multivariate outcome on infection a-ORs = odds ratios adjusted (a) for factors in Table 1, and age of the child. Bold face type indicates statistical significance with a p-value < 0.05

### Are the associations between breastfeeding and reported infections modified by sex of child, place of residence or season?

Breastfeeding or length of breastfeeding and the association with infections after 6 months of age were not modified by gender, place of residence or season at last 3-month visit. The inverse association between breastfeeding at 3 months of age and respiratory infectious episodes with a reported fever (Table 4) (interaction, gender, *p* = 0.01; season age 3 months, *p* = 0.02), or with a reported otitis media (Table 5) (interaction, gender, *p* = 0.02, season at 3 months, *p* = 0.02) between 3 and 6 months of age were both modified by sex of child and season when the child was 3 months of age. Among girls, breastfeeding was associated with a lower odds of both respiratory infectious episodes with a reported fever (yes breastfeeding vs no breastfeeding; OR 0.66, 95% CI = 0.54–0.83) and a reported otitis media (yes breastfeeding vs. no vs no; OR 0.55, 95% CI = 0.41–0.74). No associations with these infections were seen among boys (respiratory infectious episodes with a reported fever; yes breastfeeding vs no breastfeeding; OR 0.98, 95% CI = 0.80–1.21; a reported otitis media, yes breastfeeding vs. no; OR 1.02, 95% CI = 0.77–1.35). Similarly, when the child was 3 months of age, only if the season was December to February was breastfeeding associated with decreased incidence of a respiratory infectious episodes with a reported fever (yes breastfeeding vs no breastfeeding; OR 0.56, 95% CI = 0.41–0.76) or with otitis media (yes breastfeeding vs no breastfeeding; OR 0.45, 95% CI = 0.29–0.70). At other 3-month seasons, no associations were observed (ORs > 0.77). Site or continent of residence did not modify the associations.

**Table 4:** Breastfeeding at 3 months of age on Febrile Respiratory Infections between 3 and 6 months of age

Factor	N	% Reporting febrile respiratory infection by whether or not child still breastfeeding at 3 months of age		Breastfeeding on odds of Febrile respiratory infection between 3 and 6 months of age		
		No	Yes	a-OR	95% CI	Interaction *p*-value
Gender						
Female	*N* = 3276	24.9%	20.4%	**0.66**	**0.54–0.83**	
Male	*N* = 3418	23.3%	23.9%	**0.98**	**0.80–1.21**	**0.01**
Continent						
US	*N* = 2665	19.1%	15.6%	0.80	0.62–1.03	
Europe	*N* = 4029	28.9%	26.0%	0.82	0.68–0.99	0.51
Season age 3mo						
Dec – Feb	*N* = 1595	26.9	19.5	**0.56**	**0.41–0.76**	
Mar – May	*N* = 1275	29.9	27.0	**0.85**	**0.64–1.12**	
Jun – Aug	*N* = 1672	24.0	25.6	**1.05**	**0.78–1.43**	
Sept - Nov	*N* = 1730	16.5	16.5	**0.83**	**0.59–1.16**	**0.02**

a-ORs = odds ratio are adjusted (a) for age of mother at birth, maternal education, single child, number of rooms in household, parental working and smoking status when child was 9 months of age, if the child was a first degree relative of a type 1 diabetic individual, whether daycare or social group had started at the last visit and other variable not being examined for interaction (gender, continent or season at 3 months of age). Bold face type indicates statistical significance with a *p*-value < 0.05

**Table 5:** Breastfeeding at 3 months of age on Infection of Middle Ear between 3 and 6 months of age

Factor	N	% Reporting infection of middle ear by whether or not child still breastfeeding at 3 months of age		Breastfeeding on odds of infection of middle between 3 and 6 months of age		
		No	Yes	a-OR	95%CI	Interaction *p*-value
Gender						
Female	N = 3276	14.1%	9.0%	**0.55**	**0.41–0.74**	
Male	N = 3418	12.2%	11.4%	**1.02**	**0.77–1.35**	**0.02**
Continent						
US	N = 2665	17.4%	13.6%	0.76	0.58–0.99	
Europe	N = 4029	9.1%	8.4%	0.77	0.57–1.05	0.68
Season age 3mo						
Dec – Feb	N = 1595	14.2%	7.8%	**0.45**	**0.29–0.70**	
Mar – May	N = 1275	15.6%	12.1%	**0.77**	**0.53–1.12**	
Jun – Aug	N = 1672	12.8%	13.1%	**1.00**	**0.67–1.48**	
Sept - Nov	N = 1730	10.4%	8.1%	**0.85**	**Few infections**	**0.02**

a-ORs = odds ratio are adjusted (a) for age of mother at birth, maternal education, single child, number of rooms in household, parental working and smoking status when child was 9 months of age, if the child was a first degree relative of a type 1 diabetic individual, whether daycare or social group had started at the last visit and other variable not being examined for interaction (gender, continent or season at 3 months of age). Bold face type indicates statistical significance with a *p*-value < 0.05

## Discussion

To better understand the results of this study, it is useful to place them in the broader context of previous research. One of the more widely published findings on the relationship between breastfeeding and concurrent illness is the decreased incidence of otitis media in children who breastfeed when compared to children who do not. The findings of this study – that both exclusive and non-exclusive breastfeeding are protective against acute otitis media – therefore echo the findings of multiple other studies [5, 6, 8]. This study further clarifies these relationships by indicating that the protective effects of breastfeeding remain at work at least through the age of 18 months for children who continue to receive breastmilk. This is not an unprecedented discovery as, according to a meta-analysis published in 2015, cumulative evidence supports that breastfeeding protects against otitis media until the age of 2 years [36].

Our findings regarding the lower rates of infective gastroenteritis in breastfed children also help to clarify relationships shown in other studies. For example, several studies found exclusive breastfeeding for the first 6 months of life to be protective against gastrointestinal infections during that time, compared to children who were formula fed or who were breastfed for a shorter period of time [9–11]. A study conducted by Dewey and colleagues, alternatively, found that children for whom breast milk was the primary source of milk up to or beyond the age of 12 months had fewer gastrointestinal infections in the first year of life than children who were never breastfed [8]. Our study helpfully further elucidates the relationship between breastfeeding and concurrent gastrointestinal illness, demonstrating that both exclusive and non-exclusive breastfeeding may offer protection against gastrointestinal illness in the first 6 months of life. This study also followed these trends through the age of 18 months, showing that the protective properties of breastfeeding continue through this age range in children who continue to receive breastmilk, but that the protection is somewhat less in the older age group.

Additionally, this study found exclusive and non-exclusive breastfeeding between ages 3–6 months to be protective against respiratory infections with fever. This category of illness could potentially be viewed as representing the most severe respiratory infections, leading us to consider the possibility that, although this study fails to show a decrease in total respiratory infectious episodes among breastfed infants aged 3–6 months, their respiratory infections may be less severe than those of non-breastfed infants. An unexpected finding of this study was that, although breastfeeding over the age of 6 months was found to be protective against concurrent conjunctivitis or tracheitis/laryngitis, respiratory infections in general - and especially common colds (which was the largest category of respiratory infection) - were more frequently reported in children over 6 months of age who were breastfed than in children who were not breastfed at those ages.

This last finding is not common in the published literature. A study conducted by Cushing et al. found, like we did, that the risk of upper respiratory infection increased with breastfeeding, but the association was not statistically significant [15]. Dewey et al. found no association between breastfeeding and the frequency of respiratory infections (which they claim were nearly all upper respiratory infections) in the first or second year of life, when comparing children who breastfed for 12 months or more to children who never breastfed [8]. And multiple studies actually found breastfeeding to be associated with lower risk of upper respiratory tract infections [11, 12], or acute respiratory infections in general [13, 14].

We hypothesize that the apparent positive association between breastfeeding and the common cold may not represent a true causal relationship. This leads us to consider one potential limitation of this study – namely that this study relies upon parental report for its data. Although data is collected on a regular basis from parents, parents may differ in the accuracy of their reporting, or their consideration of what constitutes a true illness. This variability may be magnified for so-called “minor” illnesses, like the common cold. Therefore, the perceived effect of breastfeeding on odds of respiratory illness could plausibly be the result of breastfeeding mothers’ hyper-vigilance in regards to noticing and/or reporting upper respiratory symptoms. A causal relationship is not outside the realm of possibility, however. The breastfeeding relationship places infants and mothers in very close proximity on a very regular basis, perhaps facilitating transmission of respiratory viruses. Alternatively, mothers of children with more frequent respiratory symptoms may choose to breast feed for longer to impart to their children perceived health benefits derived from breast milk. It is also possible, as suggested above, that respiratory illnesses in breastfeeding children tend to be less severe than those in children who do not breast feed, and therefore more frequently present as common cold, instead of manifesting as febrile illness, or otitis media.

Significantly less existing literature addresses the relationship between breastfeeding as an infant and acute illness as a young child, after the cessation of breastfeeding. Our study supports that exclusive breastfeeding for longer duration is related to a decreased incidence of otitis media once breastfeeding has stopped (i.e. beyond the age of 12 months) up to the age of 4 years. Longer duration of exclusive breastfeeding protecting against future incidence of otitis media has been suggested by other studies [25–27], though none followed this trend as far as this study did (up to 4 years of life). This is, therefore, an important finding, as protective effects of breastfeeding in relation to otitis media beyond the age of 2 years have been previously poorly supported and little studied [36]. This study showed no relationship between the duration of exclusive breastfeeding and future incidence of other types of respiratory infections, or gastrointestinal infections, thus failing to replicate previously published findings that longer duration of exclusive breastfeeding protects against future episodes of diarrhea [31], or future respiratory infections outside of otitis media [26, 27, 31, 32].

Although a thorough exploration of the mechanisms behind the health benefits of breastfeeding lies outside the scope of this paper, other publications have examined this topic in detail. Breast milk has many properties that may be protective against acute illness, including secretory IgA against microbes to which the mother has been exposed; antibacterial and antiviral agents like lactoferrin, lysozyme and certain fatty acids; numerous leukocytes; and oligosaccharides, which act as analogues of microbial epithelial receptors, and therefore decoys for potential pathogens [37–40]. The thymus of breastfed infants has been found to be larger than that of non-breastfed infants, and this has corresponded to increased expression of T cells [40–42]. Breastfed infants have also been found to have a larger number of healthy bacteria (most notably *Bifidocacteria* and *Lactobacilli*) in their gut microbiome, which in turn may have implications for producing further antimicrobial compounds, reducing intestinal permeability, competing with harmful bacteria for nutrients and binding sites, and maturing and stimulating local and systemic immune responses [39, 43, 44]. It has been noted, however, that adding even small amounts of formula to a predominately breastmilk diet, or introducing solid foods, shifts the microbiome of infants toward that of a formula-fed infant, which may help to explain the greater protective effects seen with exclusive breastfeeding when compared to non-exclusive breastfeeding, demonstrated both by this study and others [45, 46].

The strength of this study has been the relatively large number of children that were followed prospectively and regularly at three different sites within two different continents, allowing for a recording of infections from diverse populations under a common data collection protocol. This limited somewhat the possibility of recall bias and allowed us to examine the consistency of the associations across different populations. While the reporting of infections varied by continent [35], the association of breastfeeding with febrile respiratory infections, common cold, otitis media, and gastroenteritis were similar across continents. However, the protective influence of breastfeeding at 3 months for both ear infections and febrile respiratory infections between 3 to 6 years of age was seen only if the child was a girl and the 6 month visit was between December and February. This was not surprising, as incidence of respiratory infections is highest in the fall and winter [35] and young boys tend to have more infections than girls [47].

Despite the strengths of our study, there were also limitations. Parental reporting of infections may still be prone to misclassification as only symptomatic infections were captured, and there was some difficulty differentiating between ICD10 codes of acute vs chronic infections. Also, there is likely to be some selection bias as the study excluded those participating families that were not as compliant with the TEDDY protocol. As we have reported in a prior publication, poor compliance or early loss to follow-up were related to a higher proportion of single, younger mothers, and mothers with fewer working hours during pregnancy [48]. The influence of breastfeeding on parental reported infections may differ among this group of young families.

## Conclusions

In summary, this study highlights and clarifies several health-related benefits of breastfeeding, both while the child is being breastfed and (to a lesser degree) in the time period following breastfeeding cessation. Breastfeeding is demonstrated to be beneficial in infancy and early childhood in regards to certain respiratory and gastrointestinal illnesses, with reduced incidence of otitis media spanning from infancy even up to the age of 4 years for some breastfed children. These results should be weighed by families in the context of their own abilities and desires when it comes to breastfeeding, helping to inform their decision-making process.

## Data Availability

The datasets generated and analyzed during the current study will be made available in the NIDDK Central Repository at https://www.niddkrepository.org/studies/teddy

## Abbreviations

CI: Confidence interval
OR: Odds ratio
T1D: Type 1 Diabetes
TEDDY: The Environmental Determinants of Diabetes in the Young
WHO: World Health Organization
